# dsDNA from extracellular vesicles (EVs) in adult AML

**DOI:** 10.1007/s00277-020-04109-z

**Published:** 2020-05-30

**Authors:** Simona Bernardi, C. Zanaglio, M. Farina, N. Polverelli, M. Malagola, D. Russo

**Affiliations:** 1 Unit of Blood Diseases and Stem Cell Transplantation, Department of Clinical and Experimental Sciences, ASST Spedali Civili di Brescia, University of Brescia, 25123 Brescia, Italy; 2 CREA Laboratory (Centro di Ricerca Emato-Oncologica AIL), ASST Spedali Civili di Brescia, 25123 Brescia, Italy

Dear Editor,

We read with great interest the article by Kontopoulou et al. [1] describing the study of the dsDNA carried by extracellular vesicles (EVs) in pediatric acute myeloid leukemia (AML) patients. The authors described a superimposable mutational status between dsDNA extracted from EVs isolated in patients’ plasma and dsDNA extracted from primary leukemia cells. Moreover, they observed a decreasing number of EVs in patients undergoing treatment.

EVs, in particular exosomes, lately attracted a considerable interest in cancer research [2] and several data are suggesting that they play an important role also in the onco-hematologic field [3]: first, because they carry out important disease markers for leukemogenesis understanding [4, 5], second because of their diagnostic potential [6], and last, but not least, for the monitoring of the minimal residual disease [7].

We have previously demonstrated the feasibility of the leukemia-derived exosomes enrichment by an immunoselection system in chronic myeloid leukemia patients [7, 8]. Then, we explored the feasibility of this approach also in adult AML patients. We enrolled 7 AML patients and 1 healthy control (Table 1) and collected peripheral blood (PB) and bone marrow (BM) specimens from AML patients, while only PB was collected from healthy donor. Exosomes were isolated from 1 ml of plasma by SeleCTEV™ Enrichment kit (Exosomics Siena SpA). The kit allows the direct exosomal DNA (exoDNA) extraction and the isolation is based on a proprietary peptide affinity method. We isolated also PB and BM cells from all samples and performed a manually genomic DNA extraction by a commercial kit (Qiagen). Genomic DNA and exoDNA were then sequenced by next-generation sequencing (NGS) using a custom gene panel (Nimblegen, Roche Diagnostics). The sequencing run was performed on MiniSeq platform (Illumina).

**Table 1:** Clinical and biological features of AML cases and healthy control

	Case 1	Case 2	Case 3	Case 4	Case 5	Case 6	Case 7	Healthy control
Sex	M	F	F	M	F	F	F	M
Age	61 y	47 y	30 y	71 y	44 y	64 y	44 y	45 y
Disease status	Diagnosis	Relapse	CR (3 m post allo-SCT)	Diagnosis (AML post MDS)	Relapse	Relapse (post allo-SCT)	CR (pre allo-SCT)	Healthy, with no tumor history
MRD in BM (blast %)	76%	65%	0%	77%	79%	29%	0%	n.a.
ExoDNA quantity	13.4 ng/ul	10.1 ng/ul	6.3 ng/ul	16.3 ng/ul	16.6 ng/ul	9.3 ng/ul	4.6 ng/ul	6.2 ng/ul
Mutation on exoDNA (nr)	4	5	0	3	4	4	0	0
Mutation on PB DNA (nr)	4	3	0	3	4	4	0	0
Mutation on BM DNA (nr)	4	5	0	3	4	4	0	n.a.

*M*, male; *F*, female; *y*, years old; *m*, months; *allo-SCT*, allogeneic stem cell transplantation; *MDS*, myelodysplastic syndrome; *CR*, complete remission; *BM*, bone marrow; *n.a.*, not applicable

We first quantified the dsDNA extracted from circulating exosomes and observed that exoDNA quantity was consistent with the leukemic burden. In particular, the lowest quantity of exoDNA was isolated in case 7 presenting complete remission (CR) before allo-stem cell transplantation (allo-SCT), while the highest quantity of exoDNA was isolated in case 5 presenting relapse. By NGS analysis, in 5 out of 7 (71%) AML cases, we identified leukemia-specific mutations on exoDNA. They were confirmed on PB and BM genomic DNA in 4/5 cases (80%). In one case (case 2) (20%), the mutations were confirmed only on BM genomic DNA. In 2/7 (29%) AML cases, no leukemia-specific mutation was sequenced neither on exoDNA nor on PB and BM genomic DNA (Table 1). No pathologic variant was sequenced on healthy control DNA samples.

These results confirm the evidence reported by Kontopoulou et al. [1] in pediatric AML and extend the presence of the same phenomenon to adult cases of AML. In particular, Kontopoulou and colleagues reported a decrease in EV numbers in patients after treatment compared with initial diagnosis, while we experienced a decrease in exoDNA quantity in patients presenting therapy response or CR.

Altogether, these data uncover the role of leukemia-derived exosomes as active carriers of leukemia cell markers and the opportunity to consider the enrichment and study of exoDNA an additional diagnostic tool for a less invasive and more personalized management of both adult and pediatric AML patients. This is of pivotal importance in the so-called 4P medicine era [9, 10], even if it has to be confirmed on larger cohorts of patients and explored in other leukemias.

## Data Availability

Data are available by the corresponding author upon request.
